# New Epitopes for the Serodiagnosis of Human Borreliosis

**DOI:** 10.3390/microorganisms12112212

**Published:** 2024-10-31

**Authors:** Mônica E. T. Alcón-Chino, Virgínia L. N. Bonoldi, Rosa M. R. Pereira, Gilberto S. Gazeta, João P. R. S. Carvalho, Paloma Napoleão-Pêgo, Andressa M. Durans, André L. A. Souza, Salvatore G. De-Simone

**Affiliations:** 1 Center for Technological Development in Health (CDTS), National Institute of Science and Technology for Innovation in Neglected Population Diseases (INCT-IDPN), FIOCRUZ, Rio de Janeiro 21040-900, RJ, Brazil; moneli@ioc.fiocruz (M.E.T.A.-C.); joaopedrorsc@gmail.com (J.P.R.S.C.); pegopn@fiocruz.br (P.N.-P.); andressa.durans@fiocruz.br (A.M.D.); 2 Post-Graduation Program in Science and Biotechnology, Department of Molecular and Cellular Biology, Biology Institute, Federal Fluminense University, Niterói 22040-036, RJ, Brazil; 3 Clinical Hospital, Faculty of Medicine, São Paulo University, São Paulo 05403-000, SP, Brazil; virginia.bonoldi@hc.fm.usp.br; 4 Faculty of Medicine, São Paulo University, São Paulo 01246-903, SP, Brazil; reumatologia.fmusp@hc.fm.usp.br; 5 Laboratory of Ticks and Other Wingless Arthropods-National Reference for Vectors of Rickettsioses, Instituto Oswaldo Cruz-IOC, FIOCRUZ, Rio de Janeiro 21041-250, RJ, Brazil; gsgazeta@ioc.fiocruz.br; 6 Multidisciplinary Biochemistry Teaching Laboratory, UNIG, Nova Iguaçu 26260-045, RJ, Brazil; 0140028@professor.unig.edu.br; 7 Laboratory of Epidemiology and Molecular Systematics, Oswaldo Cruz Institut, FIOCRUZ, Rio de Janeiro 21040-900, RJ, Brazil

**Keywords:** epitope mapping, Brazilian borreliosis, ELISA, cross-reactivity

## Abstract

Lyme disease, a zoonotic infection caused by the bacterium *Borrelia burgdorferi*, is transmitted to humans through the bites of infected ticks. Its diagnosis primarily relies on serological methods; however, the existing borreliosis techniques have shown a variable sensitivity and specificity. Our study aimed to map IgG epitopes from five outer membrane proteins (Omp) from *B. burgdorferi* [Filament flagellar 41kD (PI1089), flagellar hook-associated protein (Q44767), Flagellar hook k2 protein (O51173), Putative Omp BURGA03 (Q44849), and 31 kDa OspA (P0CL66)] lipoprotein to find specific epitopes for the development of accurate diagnosis methods. Using the spot synthesis technique, a library of 380 peptides was constructed to identify linear B cell epitopes recognized by human IgG in response to specific *B. burgdorferi*-associated proteins. The reactivity of this epitope when chemically synthesized was then evaluated using ELISA with a panel of the patient’s sera. Cross-reactivity was assessed through data bank access and in vitro analysis. Among the 19 epitopes identified, four were selected for further investigation based on their signal intensity, secondary structure, and peptide matching. Validation was performed using ELISA, and ROC curve analysis demonstrated a sensitivity of ≥85.71%, specificity of ≥92.31, accuracy of ≥90.7, and AUC value of ≥0.91 for all peptides. Our cross-reactivity analysis demonstrated that the Burg/02/huG, Burg/03/huG, and Burg/12/huG peptides were not reactive to antibodies from patients with Leptospirosis and syphilis compared to those from the *B. burgdorferi* group. These peptides indicated an excellent performance in distinguishing between *B. burgdorferi*-infected and non-infected individuals and exhibited a neglected reactivity to antibodies in sera from patients with Leptospirosis and syphilis. These peptides are promising targets for recombinant development, potentially leading to more accurate serological tests and vaccines.

## 1. Introduction

*Borrelia burgdorferi*, a spirochete, is responsible for Lyme disease (LD), which is transmitted to the host (human or animal) through the bite of infected ticks [1,2]. Zoonotic diseases transmitted by vectors are more prevalent in regions with warm climates, and changes in climate can increase the risk of infection in humans [3]. Thus, tick-borne zoonoses remain of high importance for public health in North America, Europe, Asia, and other parts of the world [4,5,6].

In Brazil, a related form of LD was first described by Yoshinari and denominated initially as Baggio–Yoshinari syndrome (BYS), and later as Brazilian borreliosis or Brazilian Lyme-disease-like syndrome [7,8]. Although different microbiological and laboratory features and relapsing symptoms were pointed out, the initial symptoms of LD (erythema migrans, fever, headache, fatigue, and multisystemic inflammatory characteristics) were not visible in the patients [9,10], and spirochete were not isolated [10,11,12,13], serological evidence of LD was evident in different states of Brazil [11,12,13,14,15].

When untreated, systemic infections may occur, resulting in neurological complications, arthritis, chronic acrodermatitis, lymphocytoma, conjunctivitis, uveitis, joint pain, or heart palpitations [16,17]. However, before chronic systemic infection occurs, pathogen adherence to the host cell and avoiding the host humoral response through the differential expression of outer surface lipoproteins (Osps) are necessary [18,19,20]. This antigenic variability can lead to the manifestation of late-disseminated diseases such as Lyme arthritis, wherein patients can exhibit an IgG antibody response to the 31 kDa OspA and OspB for months or years [21,22]. Furthermore, these infections’ clinical signs and symptoms often overlap with those of other febrile illnesses, leading to misdiagnosis and inadequate treatment when serological test approaches are used [23].

Despite ongoing efforts, no specific and rapid serological diagnostic test exists for diagnosing borreliosis. This underscores the urgent need for improved testing methods. Yet, the pathogenesis of borreliosis remains partially understood, and current laboratory diagnostics require significant expertise to accurately interpret the results. The misdiagnosis of borreliosis can lead to inappropriate treatment, resulting in potential chronic infection characterized by a strong antibody response that does not effectively eliminate the pathogen. The early and accurate detection of Lyme disease is crucial for providing the appropriate treatment and preventing persistent infection. Direct detection of the infectious agent is often not feasible or practical, making serological testing the primary method for diagnosis [24,25].

The Centers for Disease Control and Prevention (CDC, USA) recommends a standard two-tiered (STT) serologic assay algorithm or modified two-tiered testing (MTTT) approved by the Food and Drug Administration (FDA) to establish the presence of particular antibodies and avoid false positive findings for diagnosing LD [26]. In Brazil, the use of *B. burgdorferi* sensu stricto G 39/40 of North American origin in Western blot analysis demonstrated a different pattern from that in the USA [27,28,29]. In this context, studies have shown that the pathogens causing Lyme borreliosis differ between regions [30,31]. In another study, variations in the sequence epitopes of OspA were observed when comparing pathogenic species [32].

OspA has been targeted for the Lyme disease vaccine LYMErix (SmithKline Beecham), but LYMErix was discontinued in 2002. However, the strategy for optimal efficacy vaccination with a multivalent OspA-based vaccine (VLA15), including six serotypes of OspA, may potentially prevent Lyme disease in endemic areas (the United States and Europe) [33,34,35]. This promising development in vaccine research offers hope for the prevention of Lyme disease in the future.

However, vaccine benefits may not be contemplated in other countries because of the diversity of the OspA serotypes [36]. In addition, *B. burgdorferi* serologic cross-reactivity with other bacterial and viral infections has been identified [37,38], representing a significant challenge in clinical practice and epidemiological research. This occurs when a test detects antibodies directed to similar antigens, resulting in false positive results, which result in inadequate treatment and, consequently, death, increasing costs for the health system and impacting epidemiological surveillance. Hence, developing specific cross-reaction tests is essential, and epitope mapping and the development of chimeric recombinant proteins can improve these tests’ specificity and sensitivity [39,40].

Therefore, in this study, we selected five surface-membrane-localized proteins (Q44767, O51173, Q44849, P0CL66, and PI1089) from *B. burgdorferi*. These proteins are the primary targets of the immune response, and we applied printable SPOT synthesis to map linear IgG epitopes. This research is crucial, as it provides a deeper understanding of the immune response to *B. burgdorferi*, which could significantly impact the diagnosis and treatment of Lyme disease. Membrane proteins also play important roles, acting as mediators of cell–cell communication and facilitating the identification of and response to pathogens.

O51173 and P0CL66 are flagellar proteins that play a role in motility and present characteristics such as virulence and antigenicity [41,42]. In addition, P0CL66 is an authentic outer membrane protein (Omp) A. It is highly immunogenic and can block antibody binding to other surface-exposed proteins. It is also required to ensure the effective migration, proliferation, and surveillance of the tick against the immune response of the new host [43,44]. Q44767 is a putative Omp, and BB3 (Q44849) is vital for transmitting *Borrelia* from ticks to mammals [45].

Building on the biological importance of these five Omps, four selected epitopes from the nineteen identified were evaluated using peptide-ELISA with a panel of sera from patients with borreliosis to assess the serodiagnosis potential of the peptide sequences.

## 2. Materials and Methods

### 2.1. Human Sera and Ethics Statement

Sera samples from 53 patients with Lyme-disease-like syndrome and diagnosed by Western blot and IFA were obtained from the Rheumatology Division-University of São Paulo, School of Medicine (U.S.P.) and Laboratory of Biodiversity in Entomology from Oswaldo Cruz Institute/FIOCRUZ. The study also included 31 sera samples from healthy blood bank donors (HEMORIO) from Rio de Janeiro. The Ethics in Research Committee (CEP-25836019.0.0000.5243)-UFF/FIOCRUZ approved the experiments involving human sera samples.

### 2.2. Synthesis of the Cellulose-Membrane Peptide Array

The entire sequences of *B. burgdorferi* (Q44767, P11089, O51173, Q4489, and P0CL66; UniProt http://www.uniprot.org/ (accessed on 20 November 2022) were covered with the synthesis of 15-residue-long peptides with the overlapping of 10 residues, automatically prepared on cellulose membranes (Amino-PEG 500-UC540; Intavis Bioanalytical Instruments AG, Köln, German) according to the standard SPOT synthesis protocol, using an Auto-Spot Robot ASP-222 (Intavis Bioanalytical Instruments AG, Köln, Germany) [46]. Positive peptide ([IHLVNNESSEVIVHK and GYPKDGNAFNNLDRI] (*Clostridium tetani*, spot P20 and P21), KEVPALTAVETGATN (*Poliovirus*, spots P22), and YPYDVPDYAGYPYDV (*H. influenza* virus hemagglutinin, spot P23)) controls were included, and the programming was carried out with the Multipep software (Intavis Bioanalytical Instruments AG, Köln, Germany). The entire library contained 380 peptides and 4 positive control peptides. The coupling reactions were followed by acetylation with acetic anhydride (4%, *v*/*v*) in N, N-dimethylformamide to render the peptides N-reactive during subsequent steps. After acetylation, the F-moc-protecting groups were removed by adding piperidine to make the nascent peptides reactive. This same coupling, blocking, and deprotection process added the remaining amino acids until the desired peptide was generated. After the addition of the last amino acid, the side chains of the amino acids were deprotected using a solution of dichloromethane–trifluoracetic acid–triisopropyl silane (1:1:0.05, *v*/*v*/*v*) and washed with ethanol, as described previously [47]. Membranes containing the synthetic peptides were probed immediately.

### 2.3. Screening of SPOT Membranes

SPOT membranes were washed for 10 min with TBS-T (50 mM Tris,136 mM NaCl, 2 mM KCl, and 0.05 Tween, pH 7.4) and then blocked with TBS-T (containing 1.5% bovine serum albumin, BSA) for 90 min at 8 °C under agitation. After extensive washing with TBS-T, the membranes were incubated for 12 h with a pool (*n* = 7) of the patient’s sera (1:150) in TBS-T + 0.75% BSA, and then washed again with TBS-T. They were incubated with alkaline-phosphatase-labeled IgG (anti-hum IgG 1: 5000; Sigma, St Louis, MO, USA) of goat anti-human IgG (H+L) for 1 h and then washed with TBS-T and CBS (50 mM citrate-buffer saline). Subsequently, chemiluminescent CDP-Star^®^ substrate (Cytiva, Marlborough, MA, USA) (0.25 mM) with Nitro-Bloc-II™ Enhancer (Applied Biosystems, Waltham, MA, USA) was added for 5 min to complete the reaction.

### 2.4. Scanning and Measurement of Spot Signal Intensities

Chemiluminescent signals were detected on an Odyssey FC (LI-COR Bioscience, Lincoln, NE, USA) using the same conditions previously described [48], with minor modifications. Briefly, a digital image file was generated at a resolution of 5 MP, and the signal intensities were quantified using the Total Lab TL100 (v 2009, Nonlinear Dynamics, Newcastle-Upon-Tyne, UK) software. This program has an automatic grid search for 384 spots but does not automatically identify possible epitope sequences. Microsoft Excel generated the chart and analyzed it. To be considered an epitope, the sequences of two or more positive contiguous spots had to present a signal intensity (SI) greater than or equal to 30% of the highest value obtained from the set of spots on the respective membrane. The signal intensity (SI), used as the background, was a set of negative controls spotted on each membrane.

### 2.5. Peptide Synthesis

A standard solid-phase synthesis protocol was used to prepare four single single peptides for the epitopes Burg/02/huG (QGWMARDLEGEK), Burg/03/huG, (TLGFDNEGA), Burg/13/huG (SNEDQPNN), and Burg/18/huG (DTDSSAAT) using the polyethylene glycol grafted TentaGel^®^M NH2 resin (RAPP Polymer, Tübingen, Land Baden-Württemberg, Germany) and an automated machine (MultiPep-1 CEM, Corp, Charlotte, NC, USA), as described previously [46]. The concentration of the peptides was determined by measuring the optical density using the molar extinction coefficient generated by the PROTPARAM software package [http://www.expasy.ch; Accessed on 20 November 2022]. The peptide sequence was confirmed by Matrix Assist-ed Laser Desorption Ionization Time-of-Flight (MALDI-TOF MS).

### 2.6. In House Enzyme-Linked Immunosorbent Assay (ELISA)

An in-house ELISA was performed as previously described, with minor modifications [49]. Briefly, ELISA plates were coated with 100 µL (0.75 µg/well) of synthetic peptide on coating buffer (Na_2_CO_3_-NaHCO_3_ buffer, 0.1 M, pH 9.6) overnight at 4 °C. After each incubation step, the plates were washed thrice with PBS-T washing buffer (PBS with 0.1% Tween 20, pH 7.2) and blocked (200 µL) using blocking buffer (PBS-T with 2.5% BSA) for 2 h at 37 °C. The plates were then incubated with 50 µL of borreliosis patients’ serum diluted in blocking buffer (1:50) for 1 h at 37 °C. After several washes with PBS-T, the plates were incubated for 1 h at 37 °C with 100 µL of goat anti-human IgG HRP (Cat # A0170, Sigma-Aldrich, St. Louis, MO, USA) diluted in blocking buffer (1:30,000), washed, and incubated for 15 min with One Step–TMB Ultra (Scienco, Santa Catarina, SC, Brazil) as the substrate. Absorbance was measured at 405 nm on a FlexStation 3 Microplate Reader (Molecular Devices, Sunnyvale, CA, USA). The immune response was defined as being significantly elevated when the optical density was above the defined cut-off, calculated as the mean of the negative controls multiplied by three times the standard deviation.

### 2.7. Bioinformatics Tools

Data bank searches were carried out on the database UniProt http://www.uniprot.org/ (accessed on 20 November 2023). To ascertain the location of the epitope within the 3D molecular structure of the protein (Q44767, P11089, O51173, Q44849, and P0CL66) from *B. burgdorferi*, in silico protein models were obtained using UCSF ChimeraX, Molecular Graphics System, Version 1.5. Protein models were obtained from the AlphaFold database [50]. Data bank searches for *B. burgdorferi* peptide and protein sequence homologies were performed using previously identified sequences in other organisms in the Protein Information Resource (PIR) (https://research.bioinformatics.udel.edu/peptidematch/index.jsp (accessed on 20 May 2023)) and UniProt (http://www.uniprot.org/ (accessed on 20 June 2023)) databases, respectively.

### 2.8. Statistical Analysis

ELISA tests were statistically analyzed using Med Calc software version 20.218 [51]. The statistical difference using a *t*-test was considered if *p*-value ≤ 0.05.

Initially, the outcomes for each peptide reported as a reactivity index (RI) were determined as the optical density (OD) ratio of a particular sample to the cut-off OD values for each test. All RI values were classified as positive (>1.00) or negative (<1.00). The gray zone refers to the sample’s RI value (RI ± 10%). Samples in the gray zone may have RI values near the cut-off, making determining the findings for these samples difficult.

One-way ANOVA with the Kruskal–Walli’s test was used to analyze the differences among multiple groups. *p* values < 0.005 were statistically significant.

## 3. Results

### 3.1. Identification of the Immunodominant IgG Epitopes in Borrelia burgdorferi

The Flagellar E (FlgE), outer surface protein A (OspA), flagellar filament 41 kDa protein (Flg 41 kDa), flagellar hook-associated protein 22 (Flg hook 2), and Omp BBA3 proteins were analyzed using BLASTP. Notably, the BBA3 (Q44849) and OspA (P0CL66) proteins matched with proteins from Borrelia sp., while the Q44767, P11089, and O51173 proteins exhibited similarities ranging from 39.29% to 57.4% with T. pallidum and L. interorgan (Table S1).

**Figure 1:**
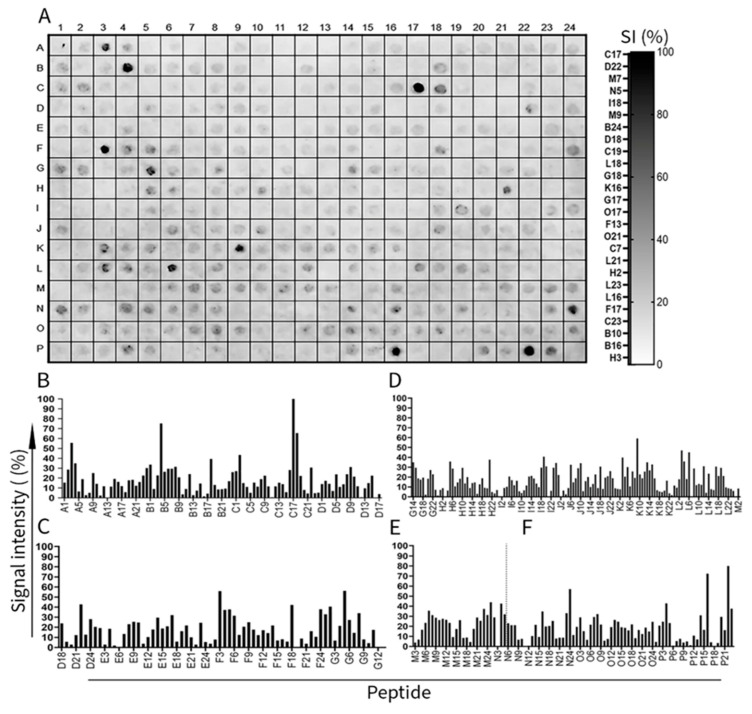
The cellulose-bound peptide library was screened, consisting of 384 peptides of 15 mer that are immunoreactive to human IgG and represent proteins from borreliosis’s flagellar and outer membranes. (**A**) Chemiluminescent assay of human IgG-reactive peptides probed with a pool of patient sera (*n* = 7) displaying the darkest spots, indicating the reactivity at each spot. Graph of the relative signal intensity (%) of human IgG reactivity at each peptide of the flagellar E protein ((**B**) Q44767), flagellar 41 kDa protein ((**C**) P11089), flagellar hook protein 2 ((**D**) O5173), BBA03 protein ((**E**) Q44849), and outer surface protein A ((**F**) P0CL66).

To map the epitopes of the Q44767, P11089, O51173, Q44849, and P0CL66 proteins, we identified the peptides recognized by the IgG antibodies in pooled sera from patients. These peptides were derived from 384 15-residue-long peptides (with 10 overlapping amino acids), as detailed in Table S2. We used serum pools containing an equivalence mixture of seven serum samples from borreliosis patients. The reaction of human IgG antibodies in sera pooled with peptides was quantified by measuring the signal intensity for each spot (Figure 1A). Absolute signals were normalized to percentages, with 100% representing the positive control. A hierarchical analysis was performed using the reactivity indices of the normalized peptides, as shown next to the nitrocellulose membrane. The reactivity was represented by an intensity scale ranging from 0% (white) to 100% (black), where darker points indicate a higher signal intensity [52]. The reactivity pattern shows variability across different peptides, with some exhibiting strong immunoreactivity (e.g., positions M7, C17, and D22), whereas others show little to no reactivity.

A ≥ 40% signal intensity threshold was set to identify IgG immunoreactive peptide sequences, identifying 19 IgG epitopes (Figure 1B). The specific peptides are listed in Table 1 for further analysis.

### 3.2. Secondary Structure and Match Peptide Analysis

The I-TASSER server supplied secondary structure prediction with an output containing the secondary sequence (H: Helix; S: Strand; C: Coil). In addition, the result of molecular modeling generated five protein models presenting values in decreasing order of C-score and TM-score. Thus, we selected the models with the highest C-score and TM-score based on the secondary structure. Table 1 depicts the information on the secondary structures of the 19 peptides and their location for each protein.

The nineteen peptide sequences were analyzed using the PIR (Protein Information Resource) peptide match. The results demonstrated that only four peptides matched with *B. burgdorferi* sp. (Table 1).

### 3.3. Localization of the Reactive Epitopes Within Flagellar and Outer Surface Proteins

**Figure 2:**
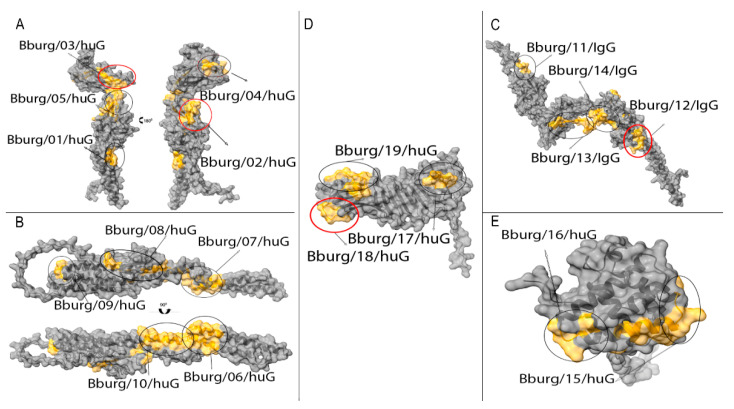
Positions of the epitopes in the 3D structures of the proteins from *B. burgdorferi*. The molecular modeling of the proteins was based on homology using AlphaFold v2.0 scrip. The selected epitopes are emphasized in orange, and four of these epitopes (represented by red circles) were chosen for evaluation using an ELISA peptide assay. (**A**) Flagellar E protein (Q44767), (**B**) flagellar 41 kDa protein (P11089), (**C**) flagellar hook protein 2 (O5173), (**D**) OspA (P0CL66) and (**E**) BBA3 protein (Q44849).

The AlphaFold Protein Structure Database provided the three-dimensional (3D) flagellar and outer surface protein structure predictions from *B. burgdorferi* [50]. The location of the B cell epitopes inserted in the 3D structure proteins were identified for each protein and are highlighted in orange, showing that all the epitopes were present on the molecular surface and, therefore, more accessible to the immune system (Figure 2).

### 3.4. Peptide Reactivity by ELISA

For this study, we selected four peptides from nineteen that showed reactivity to the IgG antibodies based on signal intensity. They had no corresponding entries in the database except for *B. burgdorferi* sp. The other fifteen epitopes presented cross-determinant sequences with various bacteria (Table 1).

**Figure 3:**
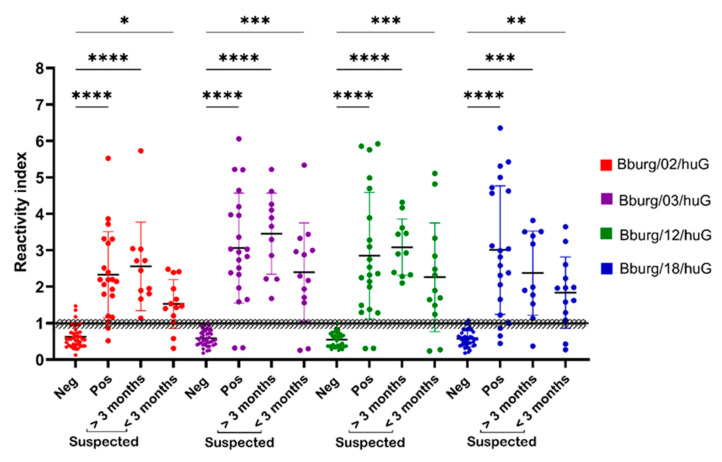
Reactivity index (RI) of synthetic peptide [Bburg/02/huG, Bburg/03/ huG, Bburg/12/huGBburg/18/huG] in individuals with anti-*Borrelia* IgG (*n* = 21), non-infected (*n* = 31), infected SC < 3 months (*n* = 13), SC > 3 months (*n* = 11). The dashed line represents the reactivity index (RI) cut-off value. The area delimited by a gray rectangle indicates the indeterminate zone (RI ± 10%). **** *p*-value < 0.0001, *** *p*-value < 0.001, ** *p*-value < 0.01, and * *p*-value ≤ 0.05. huG, reference to the IgG epitopes identified in this study by human sera.

The peptide-ELISA was tested on a panel of 21 seropositive and 39 seronegative samples, collected in periods longer than three months (*n* = 11) and less than 3 months (*n* = 13). The reactivity index (RI) and assay performance features were determined for each epitope (Figure 3). The grey zone (GZ) to the RI value was 1.0% ± 10%. We observed that the non-infected serum samples 3 (1.17%), 1 (0.39%), and 3 (1.17%) fell in the grey zone when assayed with the peptides Bburg/03/huG, Bburg/12/huG, and Bburg/18/huG, respectively. In addition, the infected serum samples were 1 (0.21%) in the GZ to both the peptides Bburg/02/huG and Bburg/18/huG; a suspected case serum sample collected in <3 months was 1 (0.13%) to the peptides Bburg/02/huG and Bburg/18/huG 1, and a suspected case serum sample collected in >3 months was 1 (0.11%) to Bburg/18/huG. The samples that fell inside the GZ were not precise enough to make a conclusive assessment.

The results presented in Table 2 show that all four peptides successfully distinguished between infected and non-infected patients, with receiver operating characteristic (ROC) values and area under the curve (AUC) scores exceeding 0.91. This indicates a high potential for differentiating positive from negative serum samples. The analysis yielded a statistically significant *p*-value of less than 0.0001, confirming the test’s strong discriminatory ability.

All four peptides recognized by the patient sera demonstrated a sensitivity above 90.48%, except for Bburg/18/huG. In terms of specificity, Bburg/03/huG and Bburg/12/huG exhibited a 100% specificity, while Bburg/02/huG and Bburg/18/huG showed a slightly lower specificity at 92.3% and 97.44%, respectively.

To confirm the peptides’ diagnostic potential, we analyzed serum samples from individuals with suspected cases exhibiting clinical symptoms consistent with borreliosis. In the serum samples collected over three months, we observed a 100% sensitivity for nearly all peptides reactive to anti-IgG, except for Bburg/18/huG, which demonstrated a 90% sensitivity (Table 2). Sensitivity decreased in the samples collected within the first three months, likely due to early IgM seroconversion and subsequent IgG seroconversion.

The specificity of the assay remained consistent across the peptides Bburg/02/huG, Bburg/03/huG, and Bburg/18/huG, with values of 92.3%, 100.0%, and 100.0%, respectively (Table 2). These specificity values were similar for both infected and suspected case groups. However, the peptide Bburg/12/huG showed variability in its specificity across the different groups. The ROC curve analysis yielded an AUC of ≥84.8 for all peptides, indicating a good accuracy and performance in the ELISA test.

### 3.5. Evaluation of Borreliosis Epitope Cross-Reactive by ELISA

BLASTP evaluation revealed a 56% identity with proteins from *Leptospira* sp. and a 51% identity with proteins from *Treponema pallidum* (syphilis). To assess the potential for cross-reactivity, we analyzed peptide/epitope interactions with sera from patients with Leptospirosis (*n* = 20) and syphilis (*n* = 20). As previously reported, these data were normalized.

**Figure 4:**
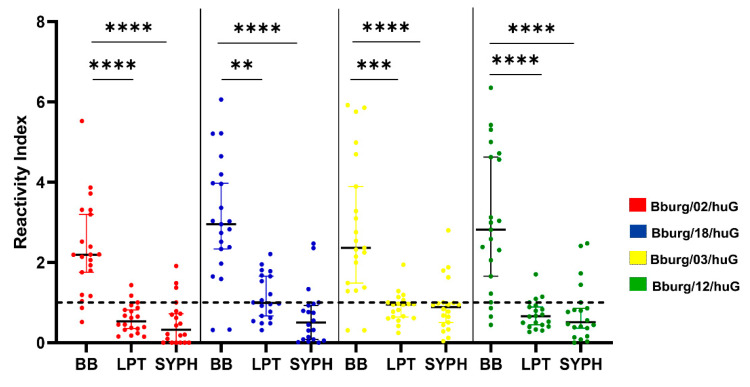
Comparison of the response to antigen in borreliosis (BB, *n* = 21), leptospirosis (LPT, *n* = 20), and syphilis (SYFH, *n* = 20) serum samples. The Kruskal–Wallis test with Dunn’s posttest was used to determine the significance of differences between groups, the *p*-value (*p* < 0.0001 (****); *p* = 0.0015 (**); *p* = 0.0001 (***).

Statistical analysis using the Kruskal–Walli’s test demonstrated a significant difference between the leptospirosis and syphilis groups compared to the borreliosis group for all peptides (Bburg/02/huG, Bburg/03/huG, Bburg/12/huG, and Bburg/18/huG), with a *p*-value of less than 0.05. Notably, the binding of Bburg/02/huG and Bburg/12/huG to antibodies in the LP and SF groups was significantly greater than that of Bburg/03/huG (BURG vs. LP: *p* = 0.0001; BURG vs. SF: *p* < 0.0001) and Burg/18/huG (BURG vs. LP: *p* = 0.0015; BURG vs. SF: *p* < 0.0001) (Figure 4).

## 4. Discussion

Borreliosis diagnosis requires further refinement in terms of sensitivity and specificity, particularly during the acute phase [27]. Research indicates that diagnosing borreliosis in Brazil is challenging due to difficulties with serological examinations, Western blot interpretations, and routine biopsies from affected individuals [6,53,54]. Studies have shown that identifying immunodominant linear IgG epitopes provides valuable information for developing passive and active immunotherapy and diagnostic tools for managing Lyme disease infection [55,56].

This study focused on outer membrane and flagellar proteins for epitope mapping due to their antigenic properties and critical role in immune responses [33,34,57]. Our results identified nineteen immunoreactive epitopes on the *Borrelia* sp. outer membrane and flagellar proteins that reacted with anti-IgG antibodies (Table 1). This finding supports the molecular identification of the hook flagellum flgE gene from *B. burgdorferi,* as presented by Mantovani et al. [58].

Numerous studies have highlighted the physicochemical properties of B cell epitopes, such as the surface accessibility of membrane-bound or free antibodies [59]. This characteristic enables antibodies to effectively bind to and neutralize their biological targets [60]. Based on predictions of the secondary and three-dimensional (3D) structures of the flagellar and outer surface proteins from *B. burgdorferi*, we found the following: (1) nineteen peptides were located in coil, helix, and strand regions (Table 1); (2) the identified B cell epitopes were exposed on the protein surfaces; (3) the peptide Burg/18/huG was localized in the C-terminal globular domain (CTD; β11−β21) of OspA [61], which has been shown to elicit protective immune responses against Lyme disease [62]; and (4) the peptides Burg/02/huG, Burg/03/huG, and Burg/12/huG were likely localized in the core domain of flagellar proteins [57].

Both the outer surface protein A (OspA) and flagellar proteins are recognized as being antigenic and immunogenic in Lyme disease [57,61,62]. OspA is one of the most abundant *B. burgdorferi* proteins and is critical in preventing infection and tissue inflammation [63]. These proteins have been exploited in vaccines and diagnostic tests for Lyme disease [34]. However, in-house ELISAs often demonstrate an inconsistent sensitivity [64,65]. The Lyme disease vaccine was also withdrawn from the market two decades ago [33]. The FDA has approved two EIA-based modified two-step testing techniques (MTTTs) to address these challenges, offering advantages over conventional testing methods, particularly in sensitivity [66].

In Brazil, studies have shown that serological tests such as ELISA and Western blotting frequently fail to meet the diagnostic criteria established by the Centers for Disease Control and Prevention (CDC) for *B. burgdorferi* infection. These limitations can lead to false negative or false positive results [67]. Nevertheless, our study demonstrates that the mapped epitopes, using sera from Brazilian patients and protein sequences obtained from international databases, effectively distinguish between healthy and infected individuals, making them promising candidates for general immunodiagnostic test development. ELISA validated this, where we observed a high sensitivity (above 90.48%) for all peptides except Bburg/18/huG. In terms of specificity, all peptides exhibited specificity levels above 92.3%. ROC curve analysis indicated an AUC of ≥ 84.8 for all peptides, suggesting a good accuracy and performance in the ELISA test.

Cross-reactivity among *Borrelia* sp. and other bacterial and viral antigens (Epstein–Barr virus, Cytomegalovirus) has been documented, particularly with *T. pallidum*, *Leptospirosis*, and *Yersinia* sp. [68,69,70,71]. No cross-reactive sequences were detected within our epitopes, and neither *Yersinia* sp., Epstein–Barr virus, nor Cytomegalovirus was identified using a data bank and blast search. However, our analysis indicated statistical significance with *p* values of < 0.005 when comparing LD patient sera with the Leptospirosis and syphilis groups. Specifically, Bburg/02/huG and Bburg/12/huG showed significantly greater differences in binding to IgG anti-Leptospirosis and anti-syphilis than IgG anti-borreliosis antibodies.

The lack of cross-reactive sequences between *Borrelia* epitopes and other bacterial or viral antigens suggests that diagnostic tools can be designed to specifically target *Borrelia* without interference from other infections, leading to more accurate diagnoses.

The demonstrated utility of peptide array screening indicates that this method can be a powerful tool for identifying unique borreliosis diagnostic peptides. This approach can facilitate the development of assays that differentiate between borreliosis and other infections, such as Leptospirosis and syphilis, which often present similar clinical symptoms.

The identified epitopes (Bburg/02/huG and Bburg/12/huG) with statistically significant differences in binding to specific IgG antibodies can not only serve as biomarkers in the development of targeted immunological assays, but also hold the promise of significantly improving diagnostic sensitivity and specificity. This potential is a reason for optimism in the field of borreliosis research.

These findings open avenues for creating multiplex diagnostic tools that simultaneously test for multiple pathogens, including *Borrelia*, while reducing false positives from cross-reactivity with other infections.

The statistical significance of the differences in IgG binding could also guide future epidemiological studies, allowing researchers to better understand the seroprevalence and co-infection patterns of borreliosis with other diseases. This underscores the significant impact this work can have on the broader field of infectious disease research.

More accurate and specific diagnostic tools will lead to better clinical management of patients suspected of having borreliosis, ensuring that appropriate treatment strategies are implemented without delay due to misdiagnosis. This highlights the potential benefits this work can provide to the healthcare community and patients.

Furthermore, ELISA tests based on whole-cell antigens have shown a low specificity, whereas some tests using recombinant antigens have demonstrated a high specificity [72,73]. Thus, as a fast and specific diagnostic test for borreliosis does not exist, screening for peptides specific to B cell epitopes is crucial in borreliosis research [74,75,76,77]. Our study identified a high sensitivity and specificity for IgG-reactive peptides, capable of discriminating between healthy and infected individuals. However, further prospective studies involving larger cohorts of individuals are necessary, since geographic variability and methodological constraints may occur.

A small cohort, as evaluated in our study, might not adequately represent the diversity of immune responses in the broader population, potentially skewing results. In addition, Lyme disease is caused by different strains of *Borrelia,* which can vary geographically. If a study primarily included samples from a specific region, the identified epitopes may not be relevant for patients from other areas where different strains predominate, despite the blast search analysis demonstrating a wide-ranging distribution of the specific epitopes.

Multiple *Borrelia* sp. and strains are associated with Lyme disease, each potentially eliciting different immune responses. If a study focused on only a few strains, it might overlook important epitopes associated with other prevalent or emerging strains.

The timing of sample collection regarding disease progression can also influence the antibody levels and the class of immunoglobulins. Suppose that samples were collected too early or too late in the infection cycle. In that case, the detected epitopes might need to reflect the same accuracy level for the development of a potent diagnostic test.

This study’s findings should ideally be validated against clinical outcomes to ensure that the identified epitopes correlate with disease severity or specific symptoms, which may have yet to be fully explored. Without a comprehensive background on the participants’ co-infections or underlying health conditions, it may be difficult to determine how these factors influence the immune response and the epitope recognition patterns.

Addressing these limitations in future studies could enhance the robustness and applicability of the findings related to specific epitopes for Lyme disease. Future research should also explore unique or chimeric epitopes, as shown by our group for the diagnosis of Chagas disease [70,78,79] or by others for that of Lyme disease [80].

In summary, these findings underscore the potential for developing more precise diagnostic tools that leverage peptide-based assays to enhance the accuracy of borreliosis diagnosis, ultimately improving patient outcomes.

## 5. Conclusions

Our study proves that selected peptides can replace the target proteins in immunodiagnostic tests for borreliosis. A peptide array can be a superior tool for screening autoantibody-based *Borrelia* biomarkers. A high-throughput array technique can economically and effectively map epitopes on a large proteome.

Nineteen IgG linear B cell epitopes were identified from different Omp and flagellar proteins. Four peptides/epitopes were selected and validated by ELISA-peptide. ROC curve analysis revealed a high sensitivity and specificity with a good accuracy in discriminating infected from non-infected individuals. We also investigated the cross-reactivity of these peptides with anti-Leptospirosis and anti-syphilis IgG antibodies. Statistical analysis of the cross reaction showed a significant difference between the Bburg/02/huG and Bburg/12/huG peptides and anti-Leptospirosis and anti-syphilis IgG antibodies compared to anti-Borreliosis IgG antibodies. The high sensitivity and specificity observed for the peptide/epitopes in this study support their use for developing specific serological tests for borreliosis infections.

## Figures and Tables

**Table 1:** B cell linear IgG epitopes screened by spot synthesis with signal intensity ≥ 40% and secondary structure. C, coil; H, helix; S, strand; * based on an I-TASSER analysis; ** PIR (Protein Information Resource).

Protein Name (Code)	Epitope Code	Location (aa)	2nd Structure *	Peptide Match **
FlgE (Q44767)	Bb/01/huG	11–25	H	Various
	Bb/02/huG	135–147	C	*B. burgdorferi* sp.
	Bb/03/huG	245–253	C	*B. burgdorferi* sp.
	Bb/04/huG	321–328	C+S	Various
	Bb/05/huG	325–336	C+S	Various
Flg41 kDa (P11089)	Bb/06/huG	121–135	C+H	Various
	Bb/07/huG	166–180	C+S	Various
	Bb/08/huG	241–244	C	Various
	Bb/09/huG	281–295	H	Various
	Bb/10/huG	296–310	H	Various
Flg Hook 2 (O51173)	Bb/11/huG	271–275	C+S	Various
	Bb/12/huG	463–470	C	*B. burgdorferi* sp.
	Bb/13/huG	546–560	S+H+C	Various
	Bb/14/huG	571–575	C+H	Various
BBA03 (Q44849)	Bb/15/huG	111–125	C+H	Various
	Bb/16/huG	130–140	C+H	Various
Osp A (P0CL66)	Bb/17/huG	56–70	C+H	Various
	Bb/18/huG	203–210	C	*B. burgdorferi* sp.
	Bb/19/huG	266–274	C+H	Various

**Table 2:** The diagnostic assessment of the peptide ELISA and receiver operating characteristic curve (ROC) with 95% confidence intervals.

	Infected				Suspected Case					
					>3 Months			<3 Months		
Peptide	Se (%)	Sp (%)	AUC	Ac (%)	Se (%)	Sp (%)	AUC	Se (%)	Sp (%)	AUC
Bburg/02/huG	90.48	92.31	0.951	90.7	100.0	92.3	0.993	84.6	92.3	0.881
Bburg/03/huG	90.48	100.0	0.915	96.6	100.0	100.0	1	84.6	100.0	0.860
Bburg/12/huG	90.48	100.0	0.912	96.5	100.0	100.0	1	84.6	100.0	0.848
Bburg/18/huG	85.71	97.44	0.949	93.1	90.9	100.0	0.933	84.6	97.3	0.877

The area under the curve (AUC), accuracy (Ac), sensitivity (Se), and specificity (Sp).

## Data Availability

The data presented in this study are available on request from the corresponding author.

## Supplementary Materials

The following supporting information can be downloaded at: https://www.mdpi.com/article/10.3390/microorganisms12112212/s1, Table S1. List of proteins used in this study and percentage sequence identity to other organism’s proteins. Table S2. List of peptides synthesized for analysis of the epitopes IgG of the five proteins of *B. burgdorferi.* [UniProt protein code 44767-Flagelar hoot ptn (spot A1-D15)]; [UniProt code P11089-Filamento flagellar 41 kDa (spot D18-G11)]; (UniProt code O51173-Flagelar hoot protein 2 (spot G14-L24); [UniProt code Q4489-Putative Omp BURGA03 (spot M3-N10)]; [UniProt code P0CL66-Omp A (spot N13-P17)].

## References

[1] Radolf J.D., Strle K., Lemieux J.E., Strle F. (2021). Lyme disease in humans. Curr. Issues Mol. Biol..

[2] Nakamura S. (2020). Spirochete Flagella and Motility. Biomolecules.

[3] Dantas-Torres F., Figueredo L.A., Sales K.G.D.S., Miranda D.E.O., Alexandre J.L.A., da Silva Y.Y., da Silva L.G., Valle G.R., Ribeiro V.M., Otranto D. (2020). Prevalence and incidence of vector-borne pathogens in unprotected dogs in two Brazilian regions. Parasit. Vectors.

[4] Rochlin I., Toledo A. (2020). Emerging tick-borne pathogens of public health importance: A mini-review. J. Med. Microbiol..

[5] Rosenberg R., Lindsey N.P., Fischer M., Gregory C.J., Hinckley A.F., Mead P.S., Paz-Bailey G., Waterman S.H., Drexler N.A., Kersh G.J. (2018). Vital signs: Trends in reported vectorborne disease cases-United States and territories, 2004–2016. MMWR Morb. Mortal. Wkly. Rep..

[6] Le Dortz L.L., Rouxel C., Polack B., Boulouis H.J., Lagrée A.C., Deshuillers P.L., Haddad N. (2024). Tick-borne diseases in Europe: Current prevention, control tools and the promise of aptamers. Vet. Parasitol..

[7] Meriläinen L., Herranen A., Schwarzbach A., Gilbert L. (2015). Morphological and biochemical features of *Borrelia burgdorferi* pleomorphic forms. Microbiology.

[8] Yoshinari N.H., Steere A.C., Cossermelli W. (1989). A review of Lyme disease. AMB Rev. Assoc. Med. Bras..

[9] Steere A.C., Malawista S.E., Hardin J.A., Ruddy S., Askenase W., Andiman W.A. (1977). Erythema chronicum migrans and Lyme arthritis. The enlarging clinical spectrum. Ann. Intern. Med..

[10] Rudenko N., Golovchenko M., Grubhoffer L., Oliver J.H. (2011). Updates on *Borrelia burgdorferi* sensu lato complex with respect to public health. Ticks Tick Borne Dis..

[11] Faccini-Martínez Á., Muñoz-Leal S., Labruna M.B., Angerami R.N. (2021). Borrelioses in Brazil: Is it time to consider tick-borne relapsing fever a neglected disease in Brazil?. Rev. Soc. Bras. Med. Trop.

[12] Oliveira S.V., Oliveira K.H.C., Santos J.U.P., Gazeta G.S. (2017). Geographical distribution of Lyme-like borreliosis in Brazil: Hot spots for research and surveillance. J. Parasit. Dis. Diagn. Ther..

[13] Carranza-Tamayo C.O., Costa J.N., Bastos W.M. (2012). Lyme disease in Tocantins, Brazil: Report of the first cases. Braz. J. Infect. Dis..

[14] Santos M., Ribeiro-Rodrigues R., Lobo R., Talhari S. (2010). Antibody reactivity to *Borrelia burgdorferi* sensu stricto antigens in patients from the Brazilian Amazon region with skin diseases not related to Lyme disease. Int. J. Dermatol..

[15] Santos C.A.D., Suzin A., Vogliotti A., Nunes P.H., Barbieri A.R.M., Labruna M.B., Szabó M.P.J., Yokosawa J. (2020). Molecular detection of a *Borrelia* sp. in nymphs of *Amblyomma brasiliense* ticks (Acari: Ixodidae) from Iguaçu National Park, Brazil, genetically related to Borrelia from Ethiopia and Côte d’Ivoire. Ticks Tick Borne Dis..

[16] Shapiro E.D. (2014). *Borrelia burgdorferi* (Lyme disease). Pediatr. Rev..

[17] Steere A.C., Strle F., Wormser G.P., Hu L.T., Branda J.A., Hovius J.W., Li X., Mead P.S. (2016). Lyme borreliosis. Nat. Rev. Dis. Primers.

[18] Coburn J., Leong J., Chaconas G. (2013). Illuminating the roles of the *Borrelia burgdorferi* adhesins. Trends Microbiol..

[19] Stone B.L., Brissette C.A. (2017). Host immune evasion by Lyme and Relapsing fever Borreliae: Findings to lead future studies. Front. Immunol..

[20] Rana V.S., Kitsou C., Dumler J.S., Pal U. (2023). Immune evasion strategies of major tick-transmitted bacterial pathogens. Trends Microbiol..

[21] Akin E., McHugh G.L., Flavell R.A., Fikrig E., Steere A.C. (1999). The immunoglobulin (IgG) antibody response to OspA and OspB correlates with severe and prolonged Lyme arthritis and the IgG response to P35 correlates with mild and brief arthritis. Infect. Immunol..

[22] Kalish R.A., McHugh G., Granquist J., Shea B., Ruthazer R., Steere A.C. (2001). Persistence of immunoglobulin M or immunoglobulin G antibody responses to *Borrelia burgdorferi* 10–20 years after active Lyme disease. Clin. Infect. Dis..

[23] Holman R.C., Paddock C.D., Curns A.T., Krebs J.W., McQuiston J.H., Childs J.E. (2001). Analysis of risk factors for fatal Rocky Mountain Spotted Fever: Evidence for superiority of tetracyclines for therapy. J. Infect. Dis..

[24] Branda J.A., Steere A.C. (2021). Laboratory diagnosis of Lyme borreliosis. Clin. Microbiol. Rev..

[25] Guérin M., Shawky M., Zedan A., Octave S., Avalle B., Maffucci I., Padiolleau-Lefèvre S. (2023). Lyme borreliosis diagnosis: State of the art of improvements and innovations. BMC Microbiol..

[26] Alcon-Chino M.E.T., De-Simone S.G. (2022). Recent advances in the immunologic method applied to tick-borne diseases in Brazil. Pathogens.

[27] Mantovani E., Costa I.P., Gauditano G., Bonoldi V.L., Higuchi M.L., Yoshinari N.H. (2007). Description of Lyme disease-like syndrome in Brazil. Is it a new tick-borne disease or Lyme disease variation?. Braz. J. Med. Biol. Res..

[28] Faccini-Martínez Á. (2019). Risk of adverse events related to prolonged antibiotic use in patients diagnosed with Baggio-Yoshinari syndrome, or autochthonous Lyme-like disease, in Brazil. Rev. Soc. Bras. Med. Trop.

[29] Bonoldi V.L.N., Yoshinari N.H., Aniz P.A.E.A., Pereira R.M.R. (2021). Innate and Th1/Th17 adaptive immunity in acute and convalescent Brazilian borreliosis disease. Braz. J. Infect. Dis..

[30] Goren A., Viljugrein H., Rivrud I.M., Jore S., Bakka H., Vindenes Y., Mysterud A. (2023). The emergence and shift in seasonality of Lyme borreliosis in Northern Europe. Proc. Biol. Sci..

[31] Haselbeck A.H., Im J., Prifti K., Marks F., Holm M., Zellweger R.M. (2022). Serology as a tool to assess infectious disease landscapes and guide public health policy. Pathogens.

[32] Steere A.C., Gross D., Meyer A.L., Huber B.T. (2001). Autoimmune mechanisms in antibiotic treatment-resistant Lyme arthritis. J. Autoimmun..

[33] Wormser G.P. (2022). A brief history of OspA vaccines including their impact on diagnostic testing for Lyme disease. Diagn. Microbiol. Infect. Dis..

[34] Dattwyler R.J., Gomes-Solecki M. (2022). The year shaped the OspA vaccine’s outcome for human Lyme disease. NPJ Vaccines.

[35] Nayak A., Schüler W., Seidel S., Gomez I., Meinke A., Comstedt P., Lundberg U. (2020). Broadly protective multivalent OspA vaccine against Lyme borreliosis, developed based on surface shaping of the C-terminal fragment. Infect. Immunol..

[36] Comstedt P., Schüler W., Meinke A., Lundberg U. (2017). The novel Lyme borreliosis vaccine VLA15 shows broad protection against *Borrelia* species expressing six different OspA serotypes. PLoS ONE.

[37] Lantos P.M., Branda J.A., Boggan J.C., Chudgar S.M., Wilson E.A., Ruffin F., Fowler V., Auwaerter P.G., Nigrovic L.E. (2015). Poor positive predictive value of Lyme disease serologic testing in an area of low disease incidence. Clin. Infect. Dis..

[38] Wojciechowska-Koszko I., Kwiatkowski P., Sienkiewicz M., Kowalczyk M., Kowalczyk E., Dołęgowska B. (2022). Cross-reactive results in serological tests for borreliosis in patients with active viral infections. Pathogens.

[39] Signorino G., Arnaboldi P.M., Petzke M.M., Dattwyler R.J. (2014). Identification of OppA2 linear epitopes as serodiagnostic markers for Lyme disease. Clin. Vaccine Immunol..

[40] Zhou J., Chen J., Peng Y., Xie Y., Xiao Y. (2022). A promising tool in serological diagnosis: Current research progress of antigenic epitopes in infectious diseases. Pathogens.

[41] Sellek R.E., Escudero R., Gil H., Rodríguez I., Chaparro E., Pérez-Pastrana E., Vivo A., Anda P. (2002). In vitro culture of *Borrelia garinii* results in loss of flagella and decreased invasiveness. Infect. Immunol..

[42] Dessau R.B., Fryland L., Wilhelmsson P., Ekerfelt C., Nyman D., Forsberg P., Lindgren P.E. (2015). Study of a cohort of 1886 persons to determine changes in antibody reactivity to *Borrelia burgdorferi* 3 months after a tick bite. Clin. Vaccine Immunol..

[43] Fridmanis J., Bobrovs R., Brangulis K., Tārs K., Jaudzems K. (2020). Structural and functional analysis of BURGA03, *Borrelia burgdorferi* competitive advantage promoting outer surface lipoprotein. Pathogens.

[44] Caimano M.J., Iyer R., Eggers C.H., Gonzalez C., Morton E.A., Gilbert M.A., Schwartz I., Radolf J.D. (2007). Analysis of the RpoS regulon in *Borrelia burgdorferi* in response to mammalian host signals provides insight into RpoS function during the enzootic cycle. Mol. Microbiol..

[45] Gilmore R.D., Piesman J. (2000). Inhibition of *Borrelia burgdorferi* migration from the midgut to the salivary glands following feeding by ticks on OspC-immunized mice. Infect. Immunol..

[46] De-Simone S.G., Napoleão-Pêgo P., Lechuga G.C., Carvalho J.P.R.S., Gomes L.R., Cardozo S.V., Morel C.M., Provance D.W., Silva F.R. (2023). High-throughput IgG epitope mapping of tetanus neurotoxin: Implications for immunotherapy and vaccine design. Toxins.

[47] De-Simone S.G., Gomes L.R., Napoleão-Pêgo P., Lechuga G.C., de Pina J.S., da Silva F.R. (2021). Epitope mapping of the diphtheria toxin and development of an ELISA-specific diagnostic assay. Vaccines.

[48] da Silva F.R., Napoleão-Pego P., De-Simone S.G. (2014). Identification of linear B epitopes of pertactin of *Bordetella pertussis* induced by immunization with whole and acellular vaccine. Vaccine.

[49] De-Simone S.G., Souza A.L.A., Aguiar A.S., Melgarejo A.R., Provance D.W. (2017). Development of an elisa for diagnosing reactive IgE antibodies anti-therapeutic horse sera. Toxicon.

[50] Jumper J., Evans R., Pritzel A., Green T., Figurnov M., Ronneberger O., Tunyasuvunakool K., Bates R., Žídek A., Potapenko A. (2021). Highly accurate protein structure prediction with AlphaFold. Nature.

[51] MedCalc® Statistical Software; Version 20.218. https://www.filehorse.com/download-medcalc/download/.

[52] Moutsinas G., Shuaib C., Guo W., Jarvis S. (2021). Graph hierarchy: A novel framework to analyze hierarchical structures in complex networks. Sci. Rep..

[53] Luther B., Moskophidis M. (1990). Antigenic cross-reactivity between *Borrelia burgdorferi*, *Borrelia recurrentis*, *Treponema pallidum*, and *Treponema phagedenis*. Zentralbl. Bakteriol..

[54] Talhari S., de Souza Santos M.N., Talhari C., de Lima Ferreira L.C., Silva R.M., Zelger B., Massone C., Ribeiro-Rodrigues R. (2010). Borrelia Burgdorferi “sensu lato” in Brazil: Occurrence confirmed by immunohistochemistry and focus floating microscopy. Acta Trop.

[55] Levy Y., Alcalay R., Zvi A., Makdasi E., Peretz E., Noy-Porat T., Chitlaru T., Mandelboim M., Mazor O., Rosenfeld R. (2022). Immunodominant Linear B-cell epitopes of SARS-CoV-2 Spike, identified by sera from K18-hACE2 mice infected with the WT or variant viruses. Vaccines.

[56] Kouzmitcheva G.A., Petrenko V.A., Smith G.P. (2001). Identifying diagnostic peptides for Lyme disease through epitope discovery. Clin. Diagn. Lab. Immunol..

[57] Kreutzberger M.A.B., Sobe R.C., Sauder A.B., Chatterjee S., Peña A., Wang F., Giron J.A., Kiessling V., Costa T.R.D., Conticello V.P. (2022). Flagellin outer domain dimerization modulates pathogenic and soil bacteria motility from viscous environments. Nat. Commun..

[58] Mantovani E., Marangoni R.G., Gauditano G., Bonoldi V.L., Yoshinari N.H. (2012). Amplification of the flgE gene provides evidence of a Brazilian borreliosis. Rev. Inst. Med. Trop Sao Paulo.

[59] Kringelum J.V., Nielsen M., Padkjær S.B., Lund O. (2013). Structural analysis of B-cell epitopes in antibody: Protein complexes. Mol. Immunol..

[60] Polyiam K., Phoolcharoen W., Butkhot N., Srisaowakarn C., Thitithanyanont A., Auewarakul P., Hoonsuwan T., Ruengjitchatchawalya M., Mekvichitsaeng P., Roshorm Y.M. (2021). Immunodominant linear B cell epitopes in the spike and membrane proteins of SARS-CoV-2 identified by immunoinformatics prediction and immunoassay. Sci. Rep..

[61] Ding W., Huang X., Yang X., Dunn J.J., Luft B.J., Koide S., Lawson C.L. (2000). Structural identification of a key protective B-cell epitope in Lyme disease antigen OspA. J. Mol. Biol..

[62] Haque H.M.E., Ejemel M., Vance D.J., Willsey G., Rudolph M.J., Cavacini L.A., Wang Y., Mantis N.J., Weis D.D. (2022). Human B Cell epitope map of the Lyme disease vaccine antigen, OspA. ACS Infect. Dis..

[63] Vance D.J., Basir S., Piazza C.L., Willsey G.G., Haque H.M.E., Tremblay J.M., Rudolph M.J., Muriuki B., Cavacini L., Weis D.D. (2024). Single-domain antibodies reveal unique borrelicidal epitopes on the Lyme disease vaccine antigen, outer surface protein A (OspA). Infect Immun.

[64] Arnaboldi P.M., Katseff A.S., Sambir M., Dattwyler R.J. (2022). Linear peptide epitopes derived from ErpP, p35, and FlaB in the serodiagnosis of Lyme Disease. Pathogens.

[65] Leeflang M.M., Ang C.W., Berkhout J., Bijlmer H.A., Van Bortel W., Brandenburg A.H., Van Burgel N.D., Van Dam A.P., Dessau R.B., Fingerle V. (2016). The diagnostic accuracy of serological tests for Lyme borreliosis in Europe: A systematic review and meta-analysis. BMC Infect. Dis..

[66] Kobayashi T., Auwaerter P.G. (2022). Diagnostic testing for Lyme Disease. Infect. Dis. Clin. N. Am..

[67] Yoshinari N.H., Bonoldi V.L.N., Bonin S., Falkingham E., Trevisan G. (2022). The current state of knowledge on Baggio-Yoshinari syndrome (Brazilian Lyme Disease-like Illness): Chronological presentation of historical and scientific events observed over the last 30 years. Pathogens.

[68] Golkocheva-Markova E., Christova I., Stoilov R., Najdenski H. (2008). Cross-reaction between Yersinia outer membrane proteins and anti-*Borrelia* antibodies in sera of patients with Lyme disease. Clin. Microbiol. Infect..

[69] Shin S.J., Chang Y.F., Jacobson R.H., Shaw E., Lauderdale T.L., Appel M.J., Lein D.H. (1993). Cross-reactivity between *B. burgdorferi* and other spirochetes affects specificity of serotests for detection of antibodies to the Lyme disease agent in dogs. Vet. Microbiol..

[70] Magnarelli L.A., Anderson J.F., Johnson R.C. (1987). Cross-reactivity in serological tests for Lyme disease and other spirochetal infections. J. Infect. Dis..

[71] Grąźlewska W., Holec-Gąsior L. (2023). Antibody cross-reactivity in serodiagnosis of Lyme Disease. Antibodies.

[72] Molins C.R., Delorey M.J., Sexton C., Schriefer M.E. (2016). Lyme Borreliosis serology: Performance of several commonly used laboratory diagnostic tests and a large resource panel of well-characterized patient samples. J. Clin. Microbiol..

[73] Kodym P., Kurzová Z., Berenová D., Pícha D., Smíšková D., Moravcová L., Malý M. (2018). Serological diagnostics of Lyme Borreliosis: Comparison of universal and *Borrelia* species-specific tests based on whole-cell and recombinant antigens. J. Clin. Microbiol..

[74] Arnaboldi P.M., Dattwyler R.J. (2015). Cross-reactive epitopes in *Borrelia burgdorferi* p66. Clin. Vaccine Immunol..

[75] Arnaboldi P.M., Seedarnee R., Sambir M., Callister S.M., Imparato J.A., Dattwyler R.J. (2013). Outer surface protein C peptide derived from *Borrelia burgdorferi* sensu stricto as a target for serodiagnosis of early Lyme disease. Clin. Vaccine Immunol..

[76] Toumanios C., Prisco L., Dattwyler R.J., Arnaboldi P.M. (2019). Linear B cell epitopes derived from the multifunctional surface lipoprotein BURGK32 as targets for the serodiagnosis of Lyme disease. mSphere.

[77] Tokarz R., Guo C., Sanchez-Vicente S., Horn E., Eschman A., Turk S.P., Lipkin W.I., Marques A. (2024). Identification of reactive *Borrelia burgdorferi* peptides associated with Lyme disease. mBio.

[78] Napoleão-Pêgo P., Carneiro F.R.G., Durans A.M., Gomes L.R., Morel C.M., Provance D.W., De-Simone S.G. (2021). Performance assessment of a multi-epitope chimeric antigen for the serological diagnosis of acute Mayaro fever. Sci. Rep..

[79] Dias E.R., Durans A.M., Succar B.B., Teixeira-Pinto L.A.L., Lechuga G.L., Miguez M.G., Figueira-Mansur J., Argondizzo A.P.C., Bernardo A.R., Diniz R.L. (2024). A high-performance multi-epitope protein for the serodiagnosis of chronic Chagas disease in ELISA and lateral flow platforms. Int. J. Mol. Sci..

[80] O’Bier N.S., Camire A.C., Patel D.T., Billingsley J.S., Hodges K.R., Marconi R.T. (2024). Development of novel multi-protein chimeric immunogens. that protect against infection with the Lyme disease agent, *Borreliella burgdorferi*. mBio.

