# Supplementary material for: New Epitopes for the Serodiagnosis of Human Borreliosis
Source: Microorganisms. 2024 Oct 31;12(11):2212. doi: 10.3390/microorganisms12112212 (PMC11596413; doi:10.3390/microorganisms12112212)
Supplement: Supplementary file 1 [file microorganisms-12-02212-s001.zip › microorganisms-3281304-supplementary.pdf]

Table S1. List of proteins used in this study and percentage sequence identity to other organism's proteins.

UniProt IDs	Protein name	BlastP	
		Organism	Identity (%)
Q44767	FlgE	*Treponema pallidum*	51.51
		Leptospira interrogans	56.85
P11089	Flg filament 41 kDa core protein	*Treponema pallidum*	57.4
		Leptospira interrogans	39.29
O51173	Flg hook- associated protein 2	*Treponema pallidum*	50.0
Q44849	Putative outer membrane protein BBA03	Borrelia sp.	None matched
P0CL66	OSP A	Borrelia sp.	None matched

Table S1: List of peptides synthesized for analysis of the epitopes IgG from *Borrelia burgdorferi*. [protein code 4767-Flagelar hoot ptn (spot A1-D15)]; [protein code P11089-Filamento flagellar 41 kDa (spot D18-G11)]; (protein O51173-Flagelar hoot protein 2 (spot G14-L24); [protein pcode Q4489-Putative OMP BBAQ3 (spot M3-N10)]; [protein code P0CL66-OMP protein A (spot N13-P17)].

spot	Peptide sequence	D24	SSGYRINRASDDAAG	H24	GNSRFVLQSLKEGKE	L24	QENTLKAFDFNQRNK
A1	MMRSLYSGVSGLQNH	E1	INRASDDAAGMGVSG	I1	VLQSLKEGKENKLVI	M1	
A2	YSGVSGLQNHQTRMD	E2	DDAAGMGVSGKINAQ	I2	KEGKENKLVIKGEGL	M2	
A3	GLQNHQTRMDVVGNN	E3	MGVSGKINAQIRGLS	I3	NKLVIKGEGLSFAKQ	M3	MKKTIIVFIILAFML
A4	QTRMDVVGNNIANVN	E4	KINAQIRGLSQASRN	I4	KGEGLSFAKQIGILS	M4	IVFIILAFMLNCKNK
A5	VVGNNIANVNTIGFK	E5	IRGLSQASRNTSKAI	I5	SFAKQIGILSELKTN	M5	LAFMLNCKNKSNDAE
A6	IANVNTIGFKKGRVN	E6	QASRNTSKAINFIQT	I6	IGILSELKTNFNPNL	M6	NCKNKSNDAEPNNDL
A7	TIGFKKGRVNFQDMI	E7	TSKAINFIQTTEGNL	I7	ELKTNFNPNLSDIVV	M7	SNDAEPNNDLDEKSQ
A8	KGRVNFQDMISQSIS	E8	NFIQTTEGNLNEVEK	I8	FNPNLSDIVVNQSSS	M8	PNNDLDEKSQAKSNL
A9	FQDMISQSISGASRP	E9	TEGNLNEVEKVLVRM	I9	SDIVVNQSSSNNKLA	M9	DEKSQAKSNLVDEDR
A10	SQSISGASRPTDARG	E10	NEVEKVLVRMKELAV	I10	NQSSSNNKLAFENNG	M10	AKSNLVDEDRIEFSK
A11	GASRPTDARGGTNPK	E11	VLVRMKELAVQSGNG	I11	NNKLAFENNGLVLNP	M11	VDEDRIEFSKATPLE
A12	TDARGGTNPKQVGLG	E12	KELAVQSGNGTYSDA	I12	FENNGLVLNPLSEVS	M12	IEFSKATPLEKLVSR
A13	GTNPKQVGLGMNVAS	E13	QSGNGTYSDADRGSI	I13	LVLNPLSEVSIEIPE	M13	ATPLEKLVSRLNLNN
A14	QVGLGMNVASIDTIH	E14	TYSDADRGSIQIEIE	I14	LSEVSIEIPEDIEIT	M14	KLVSRLNLNNTEKET
A15	MNVASIDTIHTQGAF	E15	DRGSIQIEIEQLTDE	I15	IEIPEDIEITSRSKI	M15	LNLNNTEKETLTFLT
A16	IDTIHTQGAFQSTQK	E16	QIEIEQLTDEINRIA	I16	DIEITSRSKIKFEVK	M16	TEKETLTFLTNLLKE
A17	TQGAFQSTQKASDLG	E17	QLTDEINRIADQAQY	I17	SRSKIKFEVKYFDTG	M17	LTFLTNLLKEKLVDP
A18	QSTQKASDLGVSGNG	E18	INRIADQAQYNQMHM	I18	KFEVKYFDTGLEEPD	M18	NLLKEKLVDPNIGLH
A19	ASDLGVSGNGFFILK	E19	DQAQYNQMHMLSNKS	I19	YFDTGLEEPDSKIIF	M19	KLVDPNIGLHFKNSG
A20	VSGNGFFILKEGKNL	E20	NQMHMLSNKSASQNV	I20	LEEPDSKIIFNPGGA	M20	NIGLHFKNSGGDESK
A21	FFILKEGKNLFYTRA	E21	LSNKSASQNVRTAEE	I21	SKIIFNPGGATFKDA	M21	FKNSGGDESKIEESV
A22	EGKNLFYTRAGAFDV	E22	ASQNVRTAEELGMQP	I22	NPGGATFKDAKVESE	M22	GDESKIEESVQKFLS
A23	FYTRAGAFDVDSDRH	E23	RTAEELGMQPAKINT	I23	TFKDAKVESEDSVVD	M23	IEESVQKFLSELKED
A24	GAFDVDSDRHLVNPA	E24	LGMQPAKINTPASLS	I24	KVESEDSVVDLGSDL	M24	QKFLSELKEDEIKDL
B1	DSDRHLVNPANGMRI	F1	AKINTPASLSGSQAS	J1	DSVVDLGSDLKTPLE	N1	ELKEDEIKDLLAKIK
B2	LVNPANGMRIQGWMA	F2	PASLSGSQASWTLRV	J2	LGSDLKTPLEKKYIQ	N2	EIKDLLAKIKENKDK
B3	NGMRIQGWMARDLEG	F3	GSQASWTLRVHVGAN	J3	KTPLEKKYIQMNMVK	N3	LAKIKENKDKKEKDP
B4	QGWMARDLEGEKVIN	F4	WTLRVHVGANQDEAI	J4	KKYIQMNMVKICSKE	N4	ENKDKKEKDPEELNT
B5	RDLEGEKVINTASDI	F5	HVGANQDEAIAVNIY	J5	MNMVKICSKEGSLEL	N5	KEKDPEELNTYKSIL
B6	EKVINTASDIEDLII	F6	QDEAIAVNIYAANVA	J6	ICSKEGSLELPLINI	N6	EELNTYKSILASGFD
B7	TASDIEDLIIPIGDK	F7	AVNIYAANVANLFSG	J7	GSLELPLINISNNFE	N7	YKSILASGFDGIFNQ
B8	EDLIIPIGDKEGAKS	F8	AANVANLFSGEGAQT	J8	PLINISNNFEEVEVD	N8	ASGFDGIFNQADSKT
B9	PIGDKEGAKSTKNVT	F9	NLFSGEGAQTAQAAP	J9	SNNFEEVEVDVGALS	N9	GIFNQADSKTTLNKL
B10	EGAKSTKNVTFACNL	F10	EGAQTAQAAPVQEGV	J10	EVEVDVGALSNLEEI	N10	QADSKTTLNKLKDTI
B11	TKNVTFACNLDKRLP	F11	AQAAPVQEGVQQEGA	J11	VGALSNLEEINIENK	N11	
B12	FACNLDKRLPLIQEG	F12	VQEGVQQEGAQQPAP	J12	NLEEINIENKANNKV	N12	
B13	DKRLPLIQEGANPAD	F13	QQEGAQQPAPATAPS	J13	NIENKANNKVIVISN	N13	MKKYLLGIGLILALI
B14	LIQEGANPADIARGT	F14	QQPAPATAPSQGGVN	J14	ANNKVIVISNVEIFD	N14	LGIGLILALIACKQN

B15	ANPADIARGTWVVNK	F15	ATAPSQGGVNSPVNV	J15	IVISNVEIFDPKNRD	N15	ILALIACKQNVSSLD
B16	IARGTWVVNKSLYDS	F16	QGGVNSPVNVTTTVD	J16	VEIFDPKNRDGHLPI	N16	ACKQNVSSLDEKNSV
B17	WVVNKSLYDSFGNVS	F17	SPVNVTTTVDANTSL	J17	PKNRDGHLPINAKSF	N17	VSSLDEKNSVSVDLP
B18	SLYDSFGNVSVLELR	F18	TTTVDANTSLAKIEN	J18	GHLPINAKSFAENAK	N18	EKNSVSVDLPGEMKV
B19	FGNVSVLELRVVKDL	F19	ANTSLAKIENAIRMI	J19	NAKSFAENAKIKFDG	N19	SVDLPGEMKVLVSKE
B20	VLELRVVKDLNTPNL	F20	AKIENAIRMISDQRA	J20	AENAKIKFDGVDVER	N20	GEMKVLVSKEKNKDG
B21	VVKDLNTPNLWNATV	F21	AIRMISDQRANLGAF	J21	IKFDGVDVERDSNVI	N21	LVSKEKNKDGKYDLI
B22	NTPNLWNATVLINGE	F22	SDQRANLGAFQNRLE	J22	VDVERDSNVINDLVP	N22	KNKDGKYDLIATVDK
B23	WNATVLINGEQNSNF	F23	NLGAFQNRLESIKDS	J23	DSNVINDLVPNVTLS	N23	KYDLIATVDKLELKG
B24	LINGEQNSNFTLGFD	F24	QNRLESIKDSTEYAI	J24	NDLVPNVTLSLKKPS	N24	ATVDKLELKGTSDKN
C1	QNSNFTLGFDNEGAL	G1	SIKDSTEYAIENLKA	K1	NVTLSLKKPSSDMVE	O1	LELKGTSDKNNGSGV
C2	TLGFDNEGALASLNG	G2	TEYAIENLKASYAQI	K2	LKKPSSDMVEAKIEP	O2	TSDKNNGSGVLEGVK
C3	NEGALASLNGQPGQK	G3	ENLKASYAQIKDATM	K3	SDMVEAKIEPDYEGI	O3	NGSGVLEGVKADKSK
C4	ASLNGQPGQKGDILQ	G4	SYAQIKDATMTDEVV	K4	AKIEPDYEGIKRVLL	O4	LEGVKADKSKVKLTI
C5	QPGQKGDILQIPITF	G5	KDATMTDEVVAATTN	K5	DYEGIKRVLLDFIGA	O5	ADKSKVKLTISDDLG
C6	GDILQIPITFNVLGA	G6	TDEVVAATTNSILTQ	K6	KRVLLDFIGAYNEVL	O6	VKLTISDDLGQTTLE
C7	IPITFNVLGANVGEV	G7	AATTNSILTQSAMAM	K7	DFIGAYNEVLAEINI	O7	SDDLGQTTLEVFKED
C8	NVLGANVGEVGEQQT	G8	SILTQSAMAMIAQAN	K8	YNEVLAEINIVSSNE	O8	QTTLEVFKEDGKTLV
C9	NVGEVGEQQTVNLKL	G9	SAMAMIAQANQVPQY	K9	AEINIVSSNEDQPNN	O9	VFKEDGKTLVSKKVT
C10	GEQQTVNLKLGTVGS	G10	IAQANQVPQYVLSLL	K10	VSSNEDQPNNQKSNI	O10	GKTLVSKKVTSKDKS
C11	VNLKLGTVGSYTDSI	G11	AQANQVPQYVLSLLR	K11	DQPNNQKSNIVEELT	O11	SKKVTSKDKSSTEEK
C12	GTVGSYTDSITQFAD	G12		K12	QKSNIVEELTYLSDS	O12	SKDKSSTEEKFNEKG
C13	YTDSITQFADSSSTK	G13		K13	VEELTYLSDSQKEEA	O13	STEEKFNEKGEVSEK
C14	TQFADSSSTKAIIQD	G14	MASGFFVPGLESKYN	K14	YLSDSQKEEAYKNLG	O14	FNEKGEVSEKIITRA
C15	SSSTKAIIQDGYGMG	G15	FVPGLESKYNTKEIR	K15	QKEEAYKNLGILRSE	O15	EVSEKIITRADGTRL
C16	AIIQDGYGMGYMENY	G16	ESKYNTKEIRESMLK	K16	YKNLGILRSEFLLKN	O16	IITRADGTRLEYTGI
C17	GYGMGYMENYEIDQN	G17	TKEIRESMLKSDKAK	K17	ILRSEFLLKNLKSKL	O17	DGTRLEYTGIKSDGS
C18	YMENYEIDQNGVIVG	G18	ESMLKSDKAKIDSSF	K18	FLLKNLKSKLESIIF	O18	EYTGIKSDGSGKAKE
C19	EIDQNGVIVGIYSNG	G19	SDKAKIDSSFKKLES	K19	LKSKLESIIFKPYVT	O19	KSDGSGKAKEVLKGY
C20	GVIVGIYSNGIRRDL	G20	IDSSFKKLESLEQEK	K20	ESIIFKPYVTSDPNF	O20	GKAKEVLKGYVLEGT
C21	IYSNGIRRDLGKIAL	G21	KKLESLEQEKSAWQL	K21	KPYVTSDPNFSIINQ	O21	VLKGYVLEGTLTAEK
C22	IRRDLGKIALASFMN	G22	LEQEKSAWQLINRKI	K22	SDPNFSIINQMGVFT	O22	VLEGTLTAEKTTLVV
C23	GKIALASFMNPGGLA	G23	SAWQLINRKISTLNS	K23	SIINQMGVFTNSISS	O23	LTAEKTTLVVKEGTV
C24	ASFMNPGGLAKSGDT	G24	INRKISTLNSLAKEL	K24	MGVFTNSISSSGGLS	O24	TTLVVKEGTVTLSKN
D1	PGGLAKSGDTNFVET	H1	STLNSLAKELTSLNS	L1	NSISSSGGLSRYLRL	P1	KEGTVTLSKNISKSG
D2	KSGDTNFVETSNSGQ	H2	LAKELTSLNSPFNLM	L2	SGGLSRYLRLDEKKF	P2	TLSKNISKSGEVSVE
D3	NFVETSNSGQVRIGE	H3	TSLNSPFNLMSGNSS	L3	RYLRLDEKKFDESIR	P3	ISKSGEVSVELNDTD
D4	SNSGQVRIGETGLAG	H4	PFNLMSGNSSNSEVL	L4	DEKKFDESIRNNIDN	P4	EVSVELNDTDSSAAT
D5	VRIGETGLAGLGDIR	H5	SGNSSNSEVLTLSTR	L5	DESIRNNIDNVRELF	P5	LNDTDSSAATKKTAA
D6	TGLAGLGDIRSGVLE	H6	NSEVLTLSTRYGSKN	L6	NNIDNVRELFLYDLN	P6	SSAATKKTAAWNSGT
D7	LGDIRSGVLEMANVD	H7	TLSTRYGSKNETHKL	L7	VRELFLYDLNGDRVY	P7	KKTAAWNSGTSTLTI
D8	SGVLEMANVDLAEQF	H8	YGSKNETHKLIVDQI	L8	LYDLNGDRVYDNGIA	P8	WNSGTSTLTITVNSK
D9	MANVDLAEQFTDMIV	H9	ETHKLIVDQIASADV	L9	GDRVYDNGIAKMLGD	P9	STLTITVNSKKTKDL
D10	LAEQFTDMIVTQRGF	H10	IVDQIASADVFLSSN	L10	DNGIAKMLGDCLSPL	P10	TVNSKKTKDLVFTKE
D11	TDMIVTQRGFQANAK	H11	ASADVFLSSNFDPKK	L11	KMLGDCLSPLVASGG	P11	KTKDLVFTKENTITV
D12	TQRGFQANAKTITTS	H12	FLSSNFDPKKVTIPE	L12	CLSPLVASGGVIYNK	P12	VFTKENTITVQQYDS

D13	QANAKTITTSDQLLQ	H13	FDPKKVTIPEGDYIF	L13	VASGGVIYNKIKNYD	P13	NTITVQQYDSNGTKL
D14	TITTSDQLLQELVRL	H14	VTIPEGDYIFLVGKK	L14	VIYNKIKNYDLKIFN	P14	QQYDSNGTKLEGSAV
D15	TTSDQLLQELVRLKN	H15	GDYIFLVGKKEINVK	L15	IKNYDLKIFNQKNKV	P15	NGTKLEGSAVEITKL
D16		H16	LVGKKEINVKSNGNI	L16	LKIFNQKNKVEDYKK	P16	EGSAVEITKLDEIKN
D17		H17	EINVKSNGNIDLLVK	L17	QKNKVEDYKKKYEDR	P17	AVEITKLDEIKNALK
D18	MIINHNTSAINASRN	H18	SNGNIDLLVKDINNK	L18	EDYKKKYEDRERKVE	P18	
D19	NTSAINASRNNGINA	H19	DLLVKDINNKGKGFL	L19	KYEDRERKVEGELNT	P19	
D20	NASRNNGINAANLSK	H20	DINNKGKGFLSAKIV	L20	ERKVEGELNTLDFTV	P20	IHLVNNESSEVIVHK
D21	NGINAANLSKTQEKL	H21	GKGFLSAKIVKSDKN	L21	GELNTLDFTVKRMKD	P21	GYPKDGNAFNNLDRI
D22	ANLSKTQEKLSSGYR	H22	SAKIVKSDKNGNSRF	L22	LDFTVKRMKDQENTL	P22	KEVPALTAVETGATN
D23	TQEKLSSGYRINRAS	H23	KSDKNGNSRFVLQSL	L23	KRMKDQENTLKAFDF	P23	YPYDVPDYAGYPYDV
